# Ice as a Local Anæsthetic

**Published:** 1852-09

**Authors:** W. A. Berry

**Affiliations:** Washington, D.C.


*Ice as a local Anæsthetic.* By W. A. Berry, M. D. Washington, D. C.

Messrs. Editors,—I propose to make known to the many readers of your valuable Journal the application of a new local anæsthetic agent, which probably is not familiar to a large majority of them. This agent is applicable to but a very limited part of the frame, but its efficiency is such as to cause its use in all like cases. I refer to the local anæsthetic effect of ice in the removal of the nails of the toes or fingers. This most painful operation is disarmed of all its terrors by this simple means, and the patient witnesses it with as much composure as his operator. The agent was first made use of in the wards of M. Velpeau, during the past summer, in Paris, by one of his internes, and afterwards successfully applied by himself in a number of cases. The ice is powdered finely and mixed with a sufficient quantity of salt; next enveloped in a thin cloth, and the two phalanges of the great toe or thumb enveloped in it; the application should not be continued over five or six minutes, this time being sufficient to produce the most perfect anæsthesia. M. Velpeau proceeds with the operation in the following manner: Immediately upon removing the ice, the nail is divided in its length with a common sized bistoury from its free extremity to the root, then seizing each half successively with a strong forceps, it is removed with a moderate *jerk*. The frequent necessity for the performance of this operation, and the great pain attending it when removed under other circumstances, is sufficient to cause its universal application by the Profession. M. Velpeau directs the application of compresses of cold water to the part during the first twenty-four hours; and the simple cerate dressing for a few days is all that is required.

It may be objected that the reaction under the application is such as to prevent its use; I will simply say that of the six patients that I saw operated upon by M. Velpeau, no such accident occurred to any one of them; and to the one case in which we applied it but a few days since, (and which has suggested this communication,) we have reason to believe that the agent is free from any unhappy results.

The simplicity and efficacy of this piece of minor surgery, and the so frequent necessity of some surgical interference in these cases, has induced me to send you this communication.

